# Tissue Distribution of Parrot Bornavirus 4 (PaBV-4) in Experimentally Infected Young and Adult Cockatiels (*Nymphicus hollandicus*)

**DOI:** 10.3390/v14102181

**Published:** 2022-10-01

**Authors:** Jana Petzold, Anna Maria Gartner, Sara Malberg, Jessica Bianca Link, Bianca Bücking, Michael Lierz, Christiane Herden

**Affiliations:** 1 Institute of Veterinary Pathology, Justus Liebig University, 35392 Giessen, Germany; 2 Clinic for Birds, Reptiles, Amphibian and Fish, Justus Liebig University, 35392 Giessen, Germany

**Keywords:** orthobornaviruses, avian bornavirus, proventricular dilatation disease, Psittaciformes, pathogenesis

## Abstract

Proventricular dilatation disease (PDD) caused by parrot bornavirus (PaBV) infection is an often-fatal disease known to infect Psittaciformes. The impact of age at the time of PaBV infection on organ lesions and tissue distribution of virus antigen and RNA remains largely unclear. For this purpose, tissue sections of 11 cockatiels intravenously infected with PaBV-4 as adults or juveniles, respectively, were examined via histology, immunohistochemistry applying a phosphoprotein (P) antibody directed against the bornaviral phosphoprotein and in situ hybridisation to detect viral RNA in tissues. In both groups of adult- and juvenile-infected cockatiels, widespread tissue distribution of bornaviral antigen and RNA as well as histologic inflammatory lesions were demonstrated. The latter appeared more severe in the central nervous system in adults and in the proventriculus of juveniles, respectively. During the study, central nervous symptoms and signs of gastrointestinal affection were only demonstrated in adult birds. Our findings indicate a great role of the age at the time of infection in the development of histopathological lesions and clinical signs, and thus provide a better understanding of the pathogenesis, possible virus transmission routes, and the development of carrier birds posing a risk to psittacine collections.

## 1. Introduction

The virus family *Bornaviridae* in the order *Mononegavirales*, affecting several animal species and humans, is of growing importance mainly due to its zoonotic capacity.

Since the description of Borna disease virus 1 (BoDV-1) in horses causing neurologic disorders (Borna disease), further family members have been discovered in the last years including five virus species affecting a variety of birds. Currently a novel naming of the members of the virus family *Bornaviridae* following a uniform binomial Linnean format was introduced by the International Committee on Taxonomy of Viruses (ICTV) [1,2] and mentioned in the latest official update on bornaviruses [3,4].

In 2008, parrot bornaviruses were detected as the cause of a fatal disease known as Proventricular Dilatation Disease (PDD) in Psittaciformes [5,6]. Parrot bornavirus 2 (PaBV-2) and PaBV-4 belonging to the genus *Orthobornavirus* and the species *Orthobornavirus alphapsittaciforme* are the primary affectants resulting in PDD, causing predominantly nervous and gastrointestinal symptoms [4]. Besides the classical PDD, subclinically PaBV-infected carriers and various combinations of viral distribution, inflammatory lesions, and disease status have also been described [4,7,8].

In addition, bornaviruses are known in reptiles, water birds, and fish [9,10,11]. In 2015, the newly discovered variegated squirrel bornavirus 1 (VSBV-1) was described as the zoonotic pathogen causing fatal encephalitis in humans with Prevost’s squirrels as presumed reservoir hosts [12,13,14]. 

Shortly after this, the zoonotic capacity of BoDV-1 was also unequivocally confirmed, and more than 40 human cases have been detected [15,16,17,18,19,20,21]. For BoDV-1, the bicolored white-toothed shrew is a known reservoir host [22,23,24].

The different host scenarios for BoDV-1 and VSBV-1 are quite similar. In both, BoDV-1 [15,16,18,19] and VSBV-1 [14,25] infections, dead-end hosts, or possible spill-over hosts show a severe and lethal disease with a restriction mainly to the central nervous system (CNS) with single exceptions. In contrast, BoDV-1 or VSBV-1 infected reservoir hosts are clinically inconspicuous, showing a broad viral tissue distribution [14,26,27,28]. 

In experimentally BoDV-1 infected rats and mice, a remarkable age-related difference in viral tissue distribution and development of clinical symptoms has been described [29,30,31]. BoDV-1-infected neonatal rats develop a comparable disease and viral distribution pattern as that found in reservoir hosts. In contrast, adult rats depict a restriction of virus to the CNS and a severe non-purulent meningoencephalitis due to an underlying immune-mediated process [31,32,33].

For PaBV infections, different host status as reservoir or dead-end hosts and disease outcome are less clear than in mammalian bornaviral infections. Aside from the typical development of PDD, a variety of clinical outcomes, histopathological lesions, virus distributions, and shedding exists [4,8,31,34,35,36,37,38,39]. Furthermore, infection with different PaBV species vary in clinical outcome, disease, as well as in histopathologic lesions and virus distribution [40]. The way of PaBV transmission is still not fully understood [4]. Regarding possible horizontal infections, no transmission occurred between experimentally PaBV-4 infected and noninfected cockatiels [36]. Furthermore, experimental subcutaneous (s.c.), intramuscular (i.m.), intravenous (i.v.), or intracerebral (i.c.) PaBV-2 and PaBV-4 infections are known to produce persistent infection [4]. Skin lesions are discussed as a possible way of viral infection [41]. 

Following the intramuscular PaBV-2 infection of cockatiels, differences in PaBV antigen tissue distribution and histopathologic lesions over time have been described [39,42]. Nevertheless, no data on the age of the animals at the time of infection were presented in these studies. In our recently published work we show that the time point/age of infection has an impact on the development of different courses of infection and disease, with cockatiels that were PaBV-4-infected as adults developing clinical disease unlike those infected as juveniles [43]. Nevertheless, detailed tissue distribution of PaBV antigen and RNA in these animals needed to be further investigated.

Vertical transmission has already been suspected as virus RNA detection by RT-PCR succeeded in embryonated eggs of PaBV-infected psitaccine flocks and sun conures, respectively, and in canary bornavirus (CnBV)-infected canary birds, whereas antigen detection by immunohistochemistry remained negative [44,45]. Furthermore, virus RNA and viral antigen were demonstrated in PaBV-2 and PaBV-4 in ovo infected embryos and after experimental infection of embryonic cells and embryonated eggs of cockatiels (*Nymphicus hollandicus*) with two parrot bornavirus isolates (PaBV-4 and PaBV-2**)** [46]. However, so far, no infectious virus could be isolated in these conditions [44,45,47,48].

Current studies indicate that tissue distribution of viral RNA and antigen in PaBV-infected cockatiels is most constantly seen in the alimentary tract, central nervous system, kidney, and peripheral nervous system [31,37,49]. Nevertheless, in cockatiels of different age, viral RNA and antigen are inconstantly detectable in peripheral organs [34,50]. In our recent published work, a broad tissue distribution of viral antigen and RNA in PaBV-4 infected cockatiels occurred regardless of the age at the time of infection [43]. Thus, the aim of our study was to determine age-related courses of PaBV-4 infection in adult and juvenile cockatiels, focusing on histopathological lesions and virus distribution in order to address a potential age dependency on the previously described courses of PaBV infections.

## 2. Materials and Methods

Tissue samples of 11 cockatiels infected with PaBV-4 as adults (group 1) and 11 cockatiels infected as juveniles (group 2) were included in this study. These samples originated from a recently published study performed by Gartner et al. 2021. In this previously published study, the aforementioned 11 adult and 11 newly hatched cockatiels were inoculated intravenously with a PaBV-4-isolate. At the timepoint of infection, cockatiels of group 1 were aged between 1 to 5 years, and cockatiels of group 2 were aged between 1 to 6 days. The adult animals were paired and named A to E and the additional number 0,1 for female and 1,0 for male, with one additional female named F 0,1. Juvenile animals were named J 1 to J 11. Over a time period of 233 days, these animals were observed clinically with periodic weight control. Additionally, blood samples and crop and cloacal swabs were collected regularly to detect seroconversion via indirect immunofluorescence assay (IIFA) and excretion of viral RNA via real-time RT-PCR. Virus re-isolation was performed by infectivity assay. Animals A 0,1, A 1,0, and B 1,0 were euthanized before the defined end time point. Furthermore, animals B 0,1, D 1,0, and J 9 died. For group 1 and 2, details about the materials and methods as well as the results of RT-PCR testing, serology, clinical symptoms, and necropsy are presented in our recent work published by Gartner et al. [43]. This work was accomplished with governmental permission (file reference: GI 18/9 NR.36/2015).

In the here presented study, representative organ samples from both groups were provided for detailed histology, immunohistochemistry, and in situ hybridisation for the detection of inflammatory lesions, viral antigen, and RNA distribution. Tissue samples were fixed in 10% buffered formalin and embedded in paraffin. 

Immunohistochemistry (IHC) was performed by using the polyclonal rabbit anti-BoDV-P (p24) antibody directed against the bornaviral phosphoprotein (P) with proven cross-reactivity against PaBV-4 and other bornaviruses, as described elsewhere [34,51]. Tissue samples of PaBV-4-infected cockatiels served as a positive control. Negative controls for each slide consisted of omitting the primary antibody and using a normal rabbit control serum instead. Additionally, tissue samples of a cockatiel not infected with PaBV-4 served as a negative control. 

In situ hybridisation (ISH) was performed by using a newly designed 20zz pair RNAscope^®^ probe named V-PaBV4-N targeting 141-1121 of JX065209.1:56-1177, GenBank Accession Number GCA_001430055.1. ISH was performed according to the manufacturer’s guide (RNAscope^®^ 2.5 HD Assay—BROWN, ACDBio). ISH was performed exemplarily on the tissue of four adult-infected cockatiels (B 0,1, C 0,1, C 1,0, D 1,0) and two juvenile-infected cockatiels (J 8, J 10).

IHC- and ISH-analysis were performed by applying a semiquantitative scoring system, as used elsewhere [27]. The scoring system is shown in Table 1 and reaches from “–“ meaning no detectable signal to “++++” meaning plenty of detectable signals in IHC or ISH.

^1^ HPF = high power field.

## 3. Results

### 3.1. Clinical Symptoms (Published in Gartner et al., 2021)

Typical clinical symptoms for PDD were seen in 9/11 adult cockatiels including apathy in 6/11 (A 0,1, A 1,0, B 1,0, C 1,0, D 1,0, E 0,1), gastrointestinal symptoms in 7/11 (A 1,0, B 0,1, B 1,0, C 1,0, D 1,0, E 0,1, E 1,0), neurological symptoms in 3/11 (A 1,0, C 1,0, D 1,0), weight loss in 9/11 (A 0,1, A 1,0, B 0,1, B 1,0, C 1,0, D 1,0, E 0,1, E 1,0, F 0,1), polyuria/polydipsia in 1/11 (A 1,0), and death in 2/11 (B 0,1, D 1,0) animals.

Animals A 0,1 and B 1,0 were euthanized before the defined end time point of the study due to clinical worsening and weight loss. Animals B 0,1 and D 1,0 died after showing clinical signs.

In the cohort of juvenile animals, J 9 died after severe weight loss without showing clinical symptoms, presumably due to bacterial septicaemia. The other 10 juvenile birds showed no abnormalities or clinical symptoms. Regarding clinical symptoms and weight loss, differences between the two groups of adult and juvenile cockatiels were highly significant. Detailed clinical data are shown in our recent work published by Gartner et al. [43].

### 3.2. Necropsy (Published in Gartner et al., 2021)

In 8/11 (A 0,1, A 1,0, B 0,1, B 1,0, C 1,0, D 1,0, E 0,1, E 1,0) adult animals, proventricular dilatation was documented during necropsy. The remaining 3/11 adult animals and none of the juvenile birds revealed gross lesions. The differences between the two groups were highly significant. As for clinical symptoms, detailed data are shown in our recent work published by Gartner et al. [43].

### 3.3. RT-PCR (Published in Gartner et al., 2021)

In all 22 animals, PaBV-RNA was detected via real-time RT-PCR in pooled crop and cloacal swabs or organ samples. PaBV-RNA shedding began on average earlier in the juvenile group (group 2) than in the adult group (group 1). In pooled crop and cloacal swabs, in group 1, PaBV-RNA-shedding was confirmed between days 31–63 post-infection (dpi). Regarding group 2, more homogeneously PaBV-RNA-shedding started between 27 to 36 dpi. Juvenile bird J 8 was identified as an outlier, starting later (first detection on 52 dpi) than the rest of group 2. 

PaBV-RNA-detection succeeded in all organ samples, except that of the pectoral muscle of 6/22 animals (C 1,0, D 1,0, J 1, J 2, J 4, J 7), in the liver of 2/22 animals (C 1,0, D 1,0), and in the crop of 1/22 (C 1,0) animals. On average in both groups, the highest viral loads (lowest Ct-values) were found in the cerebrum, cerebellum, spinal cord, and adrenal gland. Detailed data are shown in our recent work published by Gartner et al. [43].

### 3.4. Serology (Published in Gartner et al., 2021)

All 22 animals seroconverted. The first anti-PaBV antibodies were detected via indirect immunofluorescence assay (IIFA) on 36 dpi in three juvenile and in three adult infected cockatiels. Juvenile bird J 8 was again identified as an outliner as for PaBV-RNA shedding, showing the latest seroconversion on 99 dpi. Detailed data are shown in our recent work published by Gartner et al. [43].

### 3.5. Virus Isolation (Published in Gartner et al., 2021)

In all 22 animals, virus isolation from organ samples was successful. Detailed data are shown in our recent work published by Gartner et al. [43].

### 3.6. Histology

All adult and juvenile cockatiels developed histologic lesions most likely associated with the PaBV-4 infection. One juvenile cockatiel (J 9) died after severe weight loss, showing histologic changes that are in addition to viral infection most likely associated with septicaemia.

In the central nervous system, the most frequent changes were non-purulent encephalitis and myelitis often accompanied by satellitosis and gliosis, respectively. 

**Figure 1:**
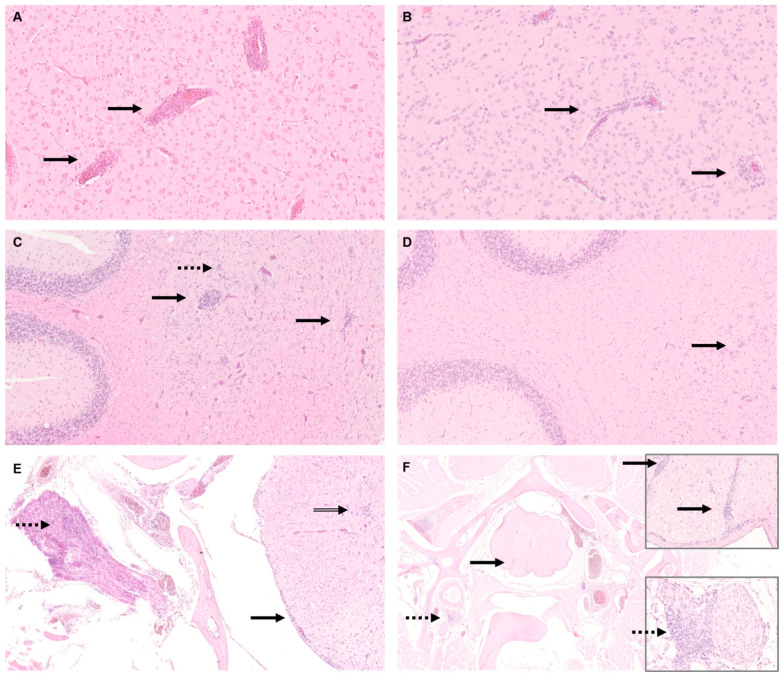
Histology of central nervous tissue of parrot bornavirus 4 (PaBV-4)-infected adult and juvenile cockatiels. (**A**) Cerebrum of adult cockatiel C 1,0. Severe non-purulent encephalitis with typical perivascular cuffs (arrow); original magnification ×200. (**B**) Cerebrum of juvenile cockatiel J 6. Moderate non-purulent encephalitis with typical perivascular cuffs (arrow) original magnification ×100. (**C**) Cerebellum of adult cockatiel D 1,0. Severe non-purulent encephalitis with perivascular cuffs (arrow) and gliosis (dashed arrow); original magnification ×100. (**D**) Cerebellum of juvenile cockatiel J6. Gliosis (arrow), no encephalitis is seen in the cerebellum in contrast to the cerebrum; original magnification ×100. (**E**) Spinal cord, spinal ganglion, and nerve of adult cockatiel D 1,0. Moderate non-purulent ganglioneuritis (dashed arrow) and moderate non-purulent meningomyelitis (arrow); original magnification ×100. (**F**) Spinal cord, spinal ganglion, and nerve of juvenile cockatiel J 6. Mild non-purulent perivascular myelitis (arrow) and severe non-purulent ganglioneuritis; original magnification ×25, magnification of inserts ×400. Hematoxylin and eosin staining (H&E).

Overall, 11/11 adult cockatiels showed alterations in the brain whereby two of them (C 1,0 and D 1,0) developed severe non-purulent encephalitis with perivascular cuffing (Figure 1A,C). In group 2, 5/11 juvenile cockatiels revealed similar but mild alterations in the brain, with one of them showing moderate encephalitis (Figure 1B,D). However, in this group, no severe alterations were noticeable. Furthermore, the brain of three adults and one juvenile cockatiel revealed non-purulent plexuschorioiditis.

In the spinal cord, 11/11 adult and 10/11 juvenile cockatiels developed mild to moderate alterations, including a non-purulent perivascular (meningo)myelitis, ganglioneuritis, satellitosis, and gliosis, respectively (Figure 1E,F).

In the eye, mild to moderate alterations were observable in 7/11 adults and 6/11 juveniles. Histologic changes in both groups varied from a non-purulent to mixed (mononuclear cells and heterophile granulocytes) conjunctivitis, with the development of lesions resembling lymphoid hyperplasia, uveitis, iridocyclitis, optic neuritis or a cell-rich *Nervus opticus*.

In the peripheral nervous system, changes were present in 4/6 adult and 2/8 juvenile animals, with mild non-purulent neuritis of the *Nervus ischiadicus* in two adults, and only minimal changes with cell-rich nerves in the remaining two adults and juveniles, respectively.

**Figure 2:**
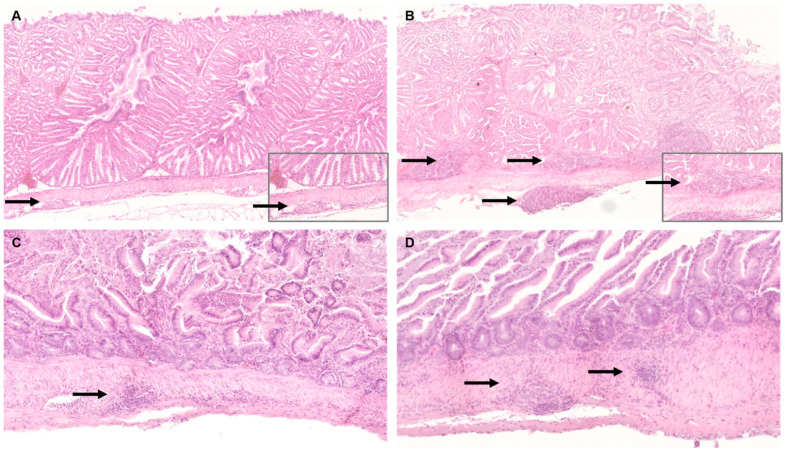
Histology of gastrointestinal tissue of Parrot bornavirus 4 (PaBV-4)-infected adult and juvenile cockatiels. (**A**) Proventriculus of adult cockatiel A 0,1. Mild non-purulent ganglionitis (arrow); original magnification x100, magnification of insert ×400. (**B**) Proventriculus of juvenile cockatiel J 2. Severe non-purulent ganglionitis (arrow), neuritis, and lymphoid hyperplasia; original magnification ×100, magnification of insert ×400. (**C**) Intestine of adult cockatiel E 1,0. Moderate ganglionitis, neuritis, and lymphoid hyperplasia (arrow) and mononuclear infiltration of the lamina propria; original magnification ×100. (**D**) Intestine of juvenile cockatiel J 6. Severe non-purulent ganglionitis, neuritis, and lymphoid hyperplasia (arrow) and mononuclear infiltration of the lamina propria; original magnification ×100; hematoxylin and eosin staining (H&E).

In the digestive system, most striking lesions were non-purulent ganglionitis, neuritis, and lymphoid hyperplasia in crop, proventriculus, gizzard, and intestine. Changes were more severe in the juvenile cockatiels than in the adults, particularly in the proventriculus (Figure 2A,B) and intestines (Figure 2C,D). Overall, 1/11 juvenile cockatiel developed severe lesions in the crop, 2/11 in the proventriculus, and 3/11 in the intestine, respectively. Furthermore, severe changes were found in the intestine of 1/11 adult cockatiel. One adult and one juvenile bird each did not reveal any histologic changes in the aforementioned organs. 

In the liver, 10/11 adults and 8/11 juveniles exhibited mild to moderate mononuclear infiltrates, mostly arranged periportally in a follicular pattern resembling lymphoid hyperplasia. Similar changes were present in the pancreas of 4/11 adults and 6/11 juveniles.

In the respiratory system, one adult cockatiel developed mixed tracheitis. The lungs revealed mononuclear infiltrates resembling lymphoid or BALT-hyperplasia

**Figure 3:**
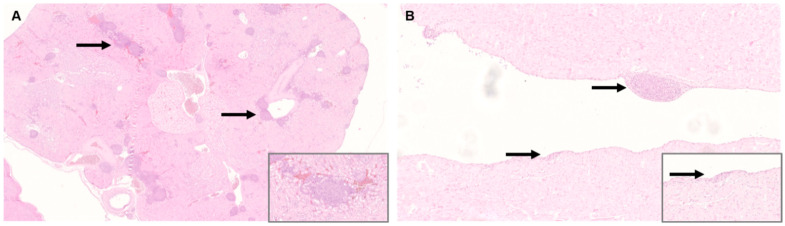
Histology of peripheral organs of Parrot bornavirus 4 (PaBV-4) infected juvenile cockatiels. (**A**) Kidney of juvenile cockatiel J 6. Severe non-purulent interstitial nephritis and lymphoid hyperplasia; original magnification ×25, magnification of insert ×200. (**B**) Heart of juvenile cockatiel J 1. Severe subendocardial ganglioneuritis (arrow); original magnification ×100, magnification of insert ×400; hematoxylin and eosin staining (H&E).

In the urogenital system, most lesions occurred in the kidney. Changes were present in 11/11 adult and 10/11 juvenile animals with severe lesions in juveniles (3/10). Lesions consisted of non-purulent interstitial nephritis, often in association with vessels or lymphoid hyperplasia (Figure 3A). A distinction between interstitial nephritis and lymphoid hyperplasia was not always possible due to the severity of the lesions and the frequent confluent follicular arrangement of lymphatic cells.

Regarding the gonads, 7/11 adults and 6/10 juveniles revealed mild to severe (two adult females) changes with mononuclear infiltrates mostly reflecting lymphoid hyperplasia in the salpinx, testis, and to a lesser extent in the stroma of the oviduct and in adjacent tissue, respectively.

Furthermore, histologic changes were present in the adrenal gland, heart, spleen, skeletal muscle, and skin of the abdomen and chest, respectively.

In the adrenal gland, 9/9 adult and 9/9 juvenile animals each developed a mild to moderate non-purulent adrenalitis or ganglioneuritis, except one adult animal (D 1,0) that developed severe changes.

In the heart, 9/11 adult and 7/11 juvenile cockatiels exhibited a non-purulent ganglionitis ranging from minimal (C 0,1) to severe (J 1) lesions (Figure 3B).

In the spleen, 9/11 adults and 4/11 juveniles exhibited a mild to severe follicular hyperplasia reflecting a splenic immunoreaction.

In the skeletal muscle, non-purulent interstitial infiltrates were present in the 2/11 juvenile cockatiels.

In the skin, mild to moderate changes were present in 11/11 adult and 9/11 juvenile cockatiels including non-purulent dermatitis and lymphoid-like hyperplasia around vessels, feather follicles, and nerve fibres, respectively.

The histologic lesions that are most likely associated with the PaBV-4-infection in adult and juvenile cockatiels are depicted in Table 2. 

### 3.7. Immunohistochemistry for the Detection of PaBV 4 Phosphoprotein

**Figure 4:**
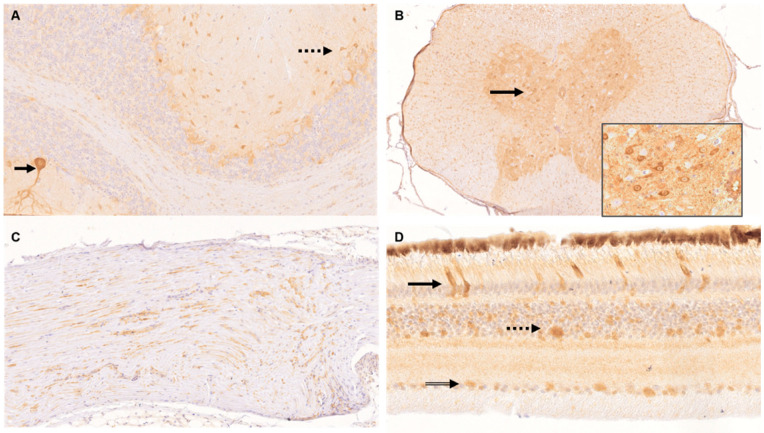
Immunohistochemistry (IHC) for the detection of viral antigen in nervous tissue of parrot bornavirus 4 (PaBV-4) infected adult and juvenile cockatiels. IHC was performed by using the phosphoprotein (P) antibody directed against the bornaviral phosphoprotein (P) with proven cross-reactivity against PaBV-4. (**A**) Cerebellum of juvenile cockatiel J 8. Demonstration of PaBV-4 P in a Purkinje cell with its dendrites (arrow) and neurons of the molecular layer (dashed arrow); original magnification ×200. (**B**) Spinal cord of juvenile cockatiel J 8. Demonstration of PaBV-4 P in neurons (arrow); original magnification ×100, magnification of insert ×400. (**C**) *Nervus ischiadicus* of adult cockatiel A 0,1. Demonstration of PaBV-4 P in single nerve fibres and endoneurium; original magnification ×200. (**D**) Retina of adult cockatiel C 0,1. Demonstration of PaBV-4 P in the photoreceptor layer (arrow), outer nuclear layer (dashed arrow) and ganglion cell layer (double lined arrow); original magnification ×400.

In the central nervous system, in the brain and spinal cord of 11/11 adult and 11/11 juvenile cockatiels, PaBV-4 antigen was present in the nucleus and cytoplasm of neurons (incl. Purkinje cells, granule cells, spinal ganglion cells), in neuronal processes, glial cells, ependymal cells, meninges, vessel walls and mononuclear perivascular cells, respectively (Figure 4A,B). 

In the peripheral nervous system, viral antigen was detected in nerve fibres, vessel walls, and endoneurium of the *Nervus ischiadicus* of 4/5 adult and 4/5 juvenile animals, respectively (Figure 4C).

In 7/11 adult and 7/10 juvenile cockatiels, tissue of the eye harboured viral antigen including the retina, conjunctiva, cornea, conjunctival glands, eyelid, iris, and ganglion cells, and nerve fibres of the optic nerve, respectively (Figure 4D).

**Figure 5:**
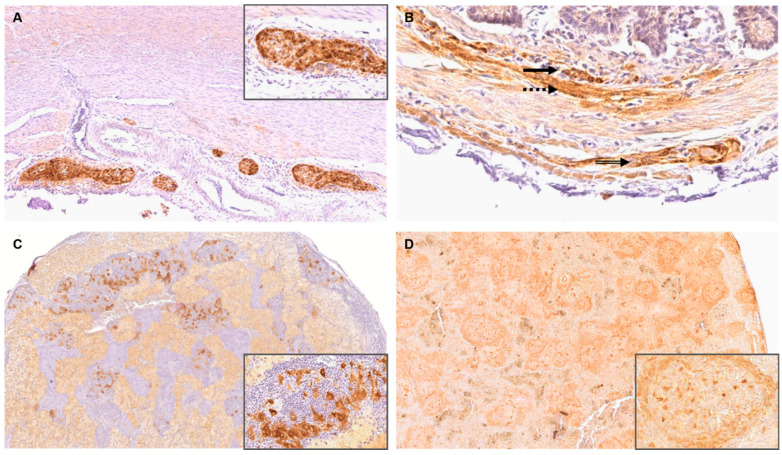
Immunohistochemistry (IHC) for the detection of viral antigen in peripheral organs of parrot bornavirus 4 (PaBV-4) infected cockatiels resembling widespread antigen distribution. IHC was performed by using the phosphoprotein (P) antibody directed against the bornaviral phosphoprotein (P) with proven cross-reactivity against PaBV-4. (**A**) Gizzard of adult cockatiel B 0,1. Demonstration of PaBV-4 P in ganglion cells and a few round cells; original magnification ×100. (**B**) Intestine of adult cockatiel B 0,1. Demonstration of PaBV-4 antigen in ganglion cells of the plexus submucosus (arrow), nerve fibres (dashed arrow) and ganglion cells of the plexus myentericus (double lined arrow); original magnification ×400. (**C**) Adrenal gland of adult cockatiel D 1,0. Demonstration of PaBV-4 P in ganglion cells; original magnification ×100, magnification of insert ×400. (**D**) Spleen of adult cockatiel C0,1. Demonstration of PaBV-4 antigen in round cells; original magnification ×100, magnification of insert ×400.

In the digestive system, antigen was present in ganglion cells, nerve fibres, smooth muscle cells, fibrocytes, vessel walls, epithelium and in mononuclear infiltrates of crop, proventriculus, gizzard, and intestine. Antigen was also demonstrable in the crop of 10/11 adult and 10/11 juvenile, in the proventriculus of 7/10 adult and 11/11 juvenile, in the gizzard of 5/8 adult and 10/11 juvenile, and in the intestine of 10/11 adult and 11/11 juvenile cockatiels, respectively (Figure 5A,B). 

In the pancreas single cells (leukocytes, reticular cells, ganglia cells, smooth muscle cells of vessel walls) exhibited PaBV-4 antigen in 1/11 adult and in 6/11 juvenile cockatiels. Demonstration of antigen in the exocrine cells remained questionable due to staining artefacts

No antigen was found in the liver of all animals examined.

In the respiratory system, PaBV-4 antigen was demonstrable in nerve fibres, vessel walls, smooth muscle cells, and fibrocytes in the trachea of 3/10 juvenile cockatiels but not in any adult animal. In the lungs, demonstration of antigen succeeded in bronchial epithelial cells, in nerve fibres, ganglion cells, fibrocytes, smooth muscle cells, and vessel walls of bronchiolar walls and septa, respectively, respiratory macrophages, type 2 pneumocytes, and mononuclear cells of BALT-hyperplasia in 7/11 adult and 8/11 juvenile cockatiels, respectively.

In the urogenital system, PaBV-4 antigen was found in the kidney of 5/11 adult and 9/10 juvenile cockatiels, respectively. Positive cell types included tubular epithelial cells, mesangial cells of glomeruli, podocytes, epithelial cells, and basement membrane of Bowman´s capsule, mononuclear cells, as well as vessel walls.

Regarding the gonads, antigen was found in tissue sections of 4/6 adult and 5/6 juvenile females and in 0/4 adult and 2/4 juvenile males. In females, immunostaining occurred in the follicular epithelium and stroma of the ovaries, in oviduct epithelium, nerve fibres, fibrocytes, smooth muscle cells, and vessel walls. In males, single cells including fibrocytes, spermatozoa and epithelial cells of seminiferous tubules harboured viral antigen.

In the adrenal gland of 7/8 adult and 8/9 juvenile birds PaBV-4, viral antigen was found mainly in ganglion cells (medulla) and epithelial cells (cortex) and to a lesser extent in mononuclear cells, vessel walls, and fibrocytes (Figure 5C).

In the spleen, viral antigen was detected in mononuclear cells with lymphocyte, macrophage, plasma cell, and reticular cell morphology, respectively, in vessel walls, smooth muscle cells, and fibrocytes of the capsule, nerve fibres, and presumably in reticular fibres, respectively, of 5/11 adult and 8/11 juvenile cockatiels (Figure 5D).

In 10/11 adult and 11/11 juvenile cockatiels, viral antigen was detectable in tissue sections of the heart in ganglion cells, nerve fibres, mononuclear cells, vessel walls, fibrocytes, and single cardiomyocytes. 

Regarding the skeletal muscle, PaBV-4 antigen was found in nerve fibres, endomysium, and vessel walls in 3/11 adult and 6/11 juvenile cockatiels. However, myocyte fibres remained negative.

Regarding the skin, skin nerve fibres, epithelium of the epidermis, and feather follicles, respectively, vessel walls, smooth muscle cells and in few cells resembling mainly lymphocytes, macrophages, and reticular cells harboured antigen in 5/11 adult and 9/11 juvenile cockatiels.

Widespread distribution of PaBV-4 phosphoprotein (PaBV-4 P) in different organ systems of both juvenile and adult cockatiels with most consistent manifestation in the central nervous system, crop, intestine, and the adrenal gland was demonstrated. An overview of infected organs and labelled cells in adult and juvenile cockatiels is given in Table 3. 

Viral antigen was demonstrated in all tissues except for the liver, which remained negative in both groups. Besides nervous tissue, fibrocytes and smooth muscle cells–often of vessel walls–harboured antigen in a wide variety of organs. As far as addressable, in vessel walls, antigen was most consistently present in smooth muscle cells and to a lower extent in fibrocytes. The detection of PaBV-4 phosphoprotein in endothelial cells of vessel walls remained questionable. Overall, antigen was present in nervous tissue as well as in cells of mesenchymal and epithelial origin. 

More detailed data of the immunohistochemical evaluation of tissues in comparison with real-time RT-PCR data that were published in Gartner et al., 2021 [43], of the adult cockatiels is given in Table S1 and of juvenile cockatiels in Table S2.

### 3.8. In Situ Hybridisation for the Detection of Viral RNA

In situ hybridisation (ISH) was performed exemplarily on tissue sections of four adult cockatiels and two juvenile cockatiels.

**Figure 6:**
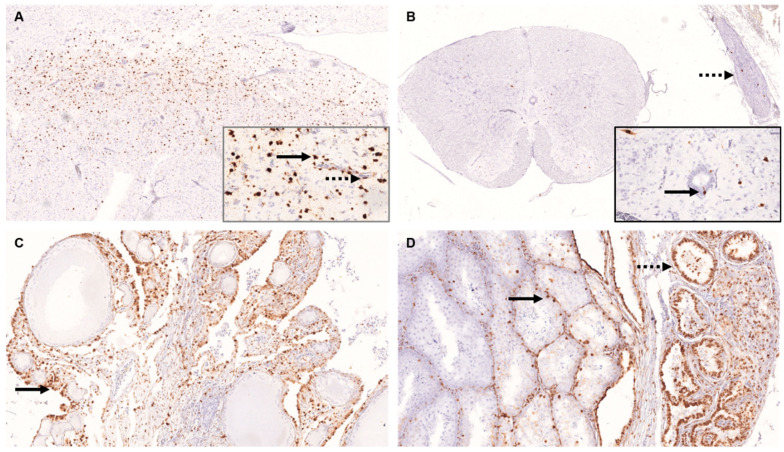
In situ hybridisation (ISH) of tissue of parrot Bornavirus 4 (PaBV-4) infected adult and juvenile cockatiels. ISH was performed by using specific RNA probes to detect sequences encoding for PaBV-4 nucleoprotein (N). (**A**) Brain of adult cockatiel D 1,0. Demonstration of PaBV-4 RNA in neurons (arrow) and endothelial cells (dashed arrow); original magnification x50, magnification of insert ×400. (**B**) Spinal cord and spinal ganglia of adult cockatiel D 1,0. Demonstration of PaBV-4 RNA in neurons, ependymal cells (arrow) and ganglion cells (dashed arrow); original magnification ×50, magnification of insert ×400. (**C**) Ovary of adult cockatiel C 0,1. Demonstration of PaBV-4 RNA in epithelial cells (arrow) and stroma; original magnification ×200. (**D**) Testis and epididymis of juvenile cockatiel J 10. Demonstration of PaBV-4 RNA in testicular spermatogenic cells (arrow) and epithelial cells of the ductus epididymis (dashed arrow); original magnification ×200.

Regarding the central nervous system (CNS), in the brain, moderate to high number of viral RNA positive cells were detected in both adult (3/3) and juvenile (1/1) cockatiels, respectively. For viral RNA positive cells in the CNS, there was a greater tendency towards the cerebrum than the cerebellum in both cohorts. Labelled cells mostly included neurons and their processes as well as glial cells, only single positive Purkinje cells were detected (Figure 6A). Furthermore, viral RNA was present in vessel walls, meninges, and leukocytes of perivascular cuffs. 

In the spinal cord, viral RNA was demonstrated in 2/3 adult and in 1/1 juvenile cockatiels in neurons, glial cells, ependymal cells, spinal ganglion cells and nerves (Figure 6B). In the juvenile cockatiel, viral RNA was additionally present in the meninges including, the arachnoid membrane, the dura mater and to a lesser extent the pia mater.

In the peripheral nervous system, no RNA was detectable in the tissue sections of the *Nervus ischiadicus* available of two adult cockatiels.

In the eye, viral RNA was identified in the epithelium and connective tissue of retina, iris, conjunctiva, and cornea, respectively of 3/4 adult and 1/1 juvenile cockatiels.

In the gastrointestinal system, tissue samples were available for ISH from two adult and one juvenile cockatiel, where RNA was detected in all tissue sections of the crop, proventriculus, gizzard, and intestine with a high number of positive cells in the proventriculus, gizzard, and intestine of one adult and in the proventriculus and gizzard of one juvenile bird, respectively. Viral RNA was detected in the neurons (ganglion cells) and nerve fibres of the plexus submucosus and myentericus, mononuclear cells, smooth muscle cells, vessel walls, and epithelial cells of all layers including the mucosa, submucosa, muscularis, and serosa, respectively.

In the liver of 1 adult and 2 juvenile cockatiels, viral RNA was present in the vessel walls and fibrocytes. Nevertheless, hepatocytes remained negative.

In the pancreas, in 1/1 adult and 2/2 juvenile cockatiels, RNA was present mainly intranuclearly in exocrine cells, fibrocytes, vessel walls, and mononuclear cells, respectively.

In the respiratory system, viral RNA was present in the tracheal epithelium in tissue available from one juvenile cockatiel.

In the lungs, RNA was detectable in the bronchial epithelium, mononuclear cells of BALT-hyperplasia, fibrocytes, smooth muscle cells, endothelial cells of vessel walls, in respiratory macrophages and type 2 pneumocytes, respectively, of 1/1 adult and 2/2 juvenile cockatiels.

In the urogenital system, viral RNA was present in the tubular epithelial cells, Bowman´s capsule and mesangium of glomeruli, mononuclear cells of lymphoid hyperplasia, fibrocytes, and vessel walls, respectively, in the kidneys of 1/1 adult and 2/2 juvenile cockatiels, with high amounts in one juvenile cockatiel (J10). 

Regarding the gonads, tissue of one adult female and two juvenile male cockatiels were available for ISH analysis. A high number of RNA-containing cells were detectable in the ovary and oviduct of the adult female with positive follicle epithelium, oviduct epithelium, fibrocytes, smooth muscle cells, and oviductal mononuclear infiltrates, respectively (Figure 6C). In the two juvenile males, a few positive cells were found in the testis, mainly in fibrocytes and smooth muscle cells, in single Sertoli cells and spermatogonia (Figure 6D).

Furthermore, PaBV-4 RNA was demonstrated in the adrenal gland, heart, spleen, skeletal muscle, and skin.

In the adrenal gland of one adult cockatiel, RNA was present in ganglion cells and mononuclear cells.

In the heart, RNA was present in tissue sections of 1/1 adult and 2/2 juvenile cockatiels in ganglion cells, nerve fibres, endocardium, vessel walls, and fibrocytes, respectively.

In the spleen, RNA was detected in mononuclear cells resembling lymphocytes, monocytes, and reticular cells, in fibrocytes, and in vessel walls in 1/1 adult and 2/2 juvenile cockatiels, respectively.

In the skeletal muscle, RNA was present in vessel walls and small nerve fibres of 1/1 adult and 1/1 juvenile cockatiel, respectively. Nevertheless, myocytes remained negative.

In the skin, RNA was present in the follicular epithelium, skin nerve fibres, dermal fibrocytes, smooth muscle cells, vessel walls, and mononuclear cells in follicular aggregates resembling lymphocytes or dermal dendritic cells, and in single cells of the epidermal epithelium, respectively, of 1/1 adult and 2/2 juvenile cockatiels.

In both, juvenile and adult cockatiels, viral RNA was demonstrable in all tissues and similar cell types. Supporting the immunohistochemical results, RNA was also present in vessel walls and fibrocytes, nevertheless, hepatocytes remained negative. As far as addressable, in vessel walls, viral RNA was present in smooth muscle cells, fibrocytes, and in endothelial cells. Overall, RNA was present in nervous tissue as well as in cells of mesenchymal and epithelial origin. 

Results are listed in Table 4 for adult and juvenile cockatiels.

## 4. Discussion

The aim of this study was to address potential age dependency in PaBV-4 infection regarding the outcome of the infection by detailing histopathologic lesions and tissue distribution of virus antigen and RNA. For this purpose, a broad range of tissues of cockatiels infected as adults or juveniles were investigated. In both cohorts of cockatiels, either PaBV-4-infected as juveniles or adults, histologic alterations were present in a broad organ spectrum including typical non-purulent inflammation in the alimentary tract and the central nervous system (CNS). In accordance with the neurotropism of PaBV, antigen and RNA were detected via IHC and ISH, respectively, in neuronal tissue, with detection in the brain tissue of adult and juvenile animals as reported elsewhere [34,42,50]. In the brain tissue of juvenile cockatiels, inflammation was less severe or absent compared to adult cockatiels that all showed mild to severe inflammatory alterations in the brain and spinal cord. 

In our previous published study, a widespread tissue distribution of PaBV-4-RNA was already confirmed by real-time RT-PCR [43]. In the here-presented study, a broad overlap of tissue distribution of viral antigen and RNA detection in tissue via IHC and ISH, respectively, could be demonstrated in accordance to the previously published results of RNA distribution detected via real-time RT-PCR. Nevertheless, some differences could be identified. For instance, in adult and juvenile PaBV-4-infected cockatiels, for PaBV-RNA, an average low Ct-values was detected in all samples of the adrenal gland [43]. In contrast, via IHC viral antigen was only demonstrated in a low number of cells on average. The exceptions were juvenile animal J 10 with a moderate Ct-value and highest numbers of viral antigen positive cells, and adult animal F 0,1 with a low Ct-value and matching demonstration of high numbers of viral antigen-positive cells. Tissue of the adrenal glands was only available in 1/6 animals for ISH, so no valid statement about the tissue RNA distribution is possible and more animals must be investigated. These discrepancies have also been reported in VSBV-1-infected squirrels and might be due an uneven viral tissue distribution or low amounts of viral antigen not detectable by IHC [27]. 

Nevertheless, in both cohorts, adult- and juvenile-PaBV-4-infected cockatiels, a widespread antigen and RNA tissue distribution was present. The widespread tissue distribution of viral antigen via IHC and RNA via ISH in possible excretion organs including alimentary tract, skin, kidneys, gonads, eye, ovary, fallopian tube, and testis indicates transmission via secretions and excretions, including feces, urine, feather dust, and vertical transmission, respectively, as reported earlier [8,34,46,50]. Furthermore, the results indicate that the virus spreads possibly not only via nerve tissue, but potentially also via endothelial cells, fibrocytes, and smooth muscle cells.

Three male adult cockatiels developed CNS symptoms during the course of infection, which can be explained by a mild to severe non-purulent encephalitis. Interestingly, neurological signs were absent in the course of infection in the juvenile cockatiels, which could be explained by the absence or only mild to moderate inflammatory alterations in brain and spinal cord, respectively. Apart from the varying severity of the lesions, an immunopathological process needs to be considered as a possible reason for different clinical outcome in both cohorts.

In contrast to the findings in the central nervous system, regarding the alimentary tract, histopathologic lesions in the proventriculus were outstanding as juvenile cockatiels developed severe histologic alterations. These findings are in contrast to the clinical signs since most adult cockatiels showed a proventricular dilatation and gastrointestinal symptoms but none of the juvenile cockatiels developed macroscopic alterations of the alimentary tract, contradicting the histopathologic lesions. Clinical data of the study are presented in our previously published work by Gartner et al. [43]. Thus, further functional alterations, possibly due to immunopathological processes, have to be considered.

The histological lesions point to an age-related course of infection with more severe lesions in the CNS of adults but marked inflammation in gastrointestinal tract of juveniles despite lacking clinical signs. Whether this could point to a kind of immune tolerance in the juveniles needs to be further investigated, as recent studies showed that vaccination with inactivated whole virus and/or immunization with viral nucleoprotein before infection could prevent from clinical signs despite viral presence [52].

Moreover, these detailed findings contribute to explain the development of carrier birds and the variability of presence of clinical signs, inflammation, and virus infection.

In both cohorts, a widespread antigen tissue distribution in adult and juvenile cockatiels was found, with most virus positive cells in the CNS and gastrointestinal tract. This also indicates an age-independent virus spread in PaBV4 infections of cockatiels and no correlation to the occurrence of clinical signs in the juveniles. Whether this could be based on a kind of immune tolerance and contribute further to the carrier status and variable disease pattern also needs further analyses. In neonatal rats, and most likely also in reservoir species of mammalian bornaviruses, a widespread viral organ distribution is associated with immune tolerance and absence or only weak inflammatory lesions [26,27,29]. In contrast, juvenile-infected cockatiels showed widespread histopathologic lesions in different organs. 

In summary, a widespread tissue distribution of viral antigen and RNA in both adult and juvenile cockatiels with differences in clinical outcome was confirmed. This contributes to understand the development of various clinical or non-clinical disease patterns and the development of carrier birds when infected as juveniles.

## 5. Conclusions

The time-point of infection, and thus, the state of the immune system plays a role in PaBV4 infection of cockatiels with age-related differences in the development of clinical symptoms. Nevertheless, widespread tissue distribution of viral antigen and RNA in both adult and juvenile PaBV-4-infected cockatiels occurred, indicating virus shedding via various routes of excretion in both juvenile and adult cockatiels. The presences of viral antigen in the testis and ovaries might support the theory of vertical transmission that needs to be further investigated.

## Figures and Tables

**Table 1:** Scoring system for immunohistochemistry and in situ hybridization.

Score	Overview in 40× Magnification in %	Overview in 400× Magnification, Mean of Cells in 5 HPFs ^1^
−	0	0
+	1–15	1–30
++	15–40	30–80
+++	40–65	80–150
++++	>65	>150

^1^ HPF = high power field.

**Table 2:** Overview of histopathologic changes in 11 adult- and 11 juvenile-PaBV-4 infected cockatiels.

Tissue	Histopathology				
	Infected as				Character of Lesions
	adults		juveniles		
	Mean score	Affected animals	Mean score	Affected animals	
**Nervous System**					
**Brain**	+(+)	11/11 *	(+)	5/11	Non-purulent encephalitis, plexuschorioiditis; satellitosis, gliosis
**Spinal cord**	+(+)	11/11	+	10/11	Non-purulent (meningo)myelitis, ganglioneuritis; satellitosis, gliosis
**N. ischiadicus**	(+)	4/6	-	2/8	Non-purulent neuritis
**Eye**	+	7/11	+	6/11	Mixed conjunctivitis, uveitis, iridocyclitis, optic neuritis, lymphoid hyperplasia
**Digestive System**					
**Crop**	+	9/11	+	8/11	Non-purulent ganglionitis, neuritis; lymphoid hyperplasia
**Proventriculus**	(+)	5/11	+(+)	8/11	
**Gizzard**	+	7/11	+	6/11	
**Intestine**	+	8/11	+(+)	8/11	
**Liver**	+	10/11	+	8/11	Non-purulent periportal infiltration
**Pancreas**	(+)	4/11	+	6/11	Non-purulent infiltration/lymphoid hyperplasia
**Respiratory System**					
**Trachea**	-	1/11	-	0/9	Mixed tracheitis
**Lung**	(+)	4/11	+	6/11	Non-purulent infiltration/BALT-hyperplasia
**Urogenital System**					
**Kidney**	+(+)	11/11	++(+)	10/11	Non-purulent interstitial nephritis/lymphoid hyperplasia, mononuclear pelvic infiltrates
**Gonads**	+	7/11	+	6/10	Non-purulent infiltration/lymphoid hyperplasia
**Other Organs**					
**Adrenal Gland**	++	9/9	+	9/9	Non-purulent adrenalitis/ganglioneuritis
**Heart**	+	9/11	+	7/11	Non-purulent ganglionitis
**Spleen**	+(+)	9/11	+	4/11	Follicular hyperplasia
**Skeletal Muscle**	-	0/11	(+)	2/11	Non-purulent perivascular interstitial infiltrates
**Skin**	+	11/11	+	9/11	Non-purulent dermatitis, lymphoid hyperplasia

* x/x = no. of positive organs/no. of available and evaluable organs from cockatiels; Scoring: +++ = severe, ++ = moderate, + = mild, - = no histologic lesions; n/a = not available or evaluable.

**Table 3:** Overview of immunohistochemical results of organs and cell types of 11 adult- and 11 juvenile-PaBV-4 infected cockatiels.

Tissue	Immunohistochemistry				
	Infected as				Labelled structures and cells
	adults		juveniles		
	Mean Score	Affected animals	Mean Score	Affected animals	
**Nervous System**					
**Brain**	++	11/11 *	(+)	11/11	Neurons (incl. spinal ganglia), neuronal processes, glial cells, meninges, vessel walls, mononuclear cells
**Spinal Cord**	++	11/11	+(+)	11/11	
**N. ischiadicus**	+	4/5	+	4/5	Nerve fibres, endoneurium, vessel walls
**Eye**	+	7/11	+	7/10	Retinal cells, conjunctival epithelium, conjunctival glands, skin follicular cells, skin nerves, corneal epithelium, fibrocytes, vessel walls, iris, mononuclear cells, ganglion cells, and nerve fibres of N. opticus
**Digestive System**					
**Crop**	+(+)	10/11	+	10/11	Ganglion cells, nerve fibres, fibrocytes, epithelial cells, smooth muscle cells of lamina muscularis, vessel walls, mononuclear cells
**Proventriculus**	+	7/10	+(+)	11/11	
**Gizzard**	+	5/8	+	10/11	
**Intestine**	+	10/11	+(+)	11/11	
**Liver**	-	0/11	-	0/11	-
**Pancreas**	-	1/11	(+)	6/11	Leukocytes, reticular cells, ganglion cells, vessel walls
**Respiratory System**					
**Trachea**	-	0/10	(+)	3/10	Nerve fibres, vessel walls, smooth muscle cells, and fibrocytes of tracheal wall
**Lung**	(+)	7/11	+	8/11	Bronchial epithelial cells, smooth muscle cells and fibrocytes bronchiolar walls, respiratory macrophages, type 2 pneumocytes, vessel walls, nerve fibres, ganglion cells, mononuclear cells
**Urogenital System**					
**Kidney**	(+)	5/11	+	9/10	Tubular cells, Bowman’s capsule and mesangium of glomeruli, mononuclear cells, fibrocytes, vessel walls
**Ovary/Oviduct**	(+)	4/6	(+)	5/6	Follicular epithelium, oviduct epithelium, nerve fibres, fibrocytes, smooth muscle cells, vessel walls
**Testes**	-	0/4	(+)	2/4	Fibrocytes, spermatozoa, epithelial cells of seminiferous tubules
**Other Organs**					
**Adrenal Gland**	+(+)	7/8	+(+)	8/9	Ganglion cells (medulla), epithelial cells (cortex), mononuclear cells, vessel walls, fibrocytes
**Heart**	(+)	10/11	+	11/11	Ganglia, nerve fibres, mononuclear cells, vessel walls, fibrocytes, cardiomyocytes
**Spleen**	(+)	5/11	+	8/11	Mononuclear cells, smooth muscle cells, fibrocytes, vessel walls, nerve fibres
**Skeletal Muscle**	-	3/11	(+)	6/11	Nerve fibres, vessel walls, fibrocytes
**Skin**	(+)	5/11	+	9/11	Follicular epithelium, skin nerve fibres, smooth muscle cells, fibrocytes, mononuclear cells, epidermal epithelium

* x/x = no. of positive organs/no. of available and evaluable organs from cockatiels; Scoring system applicated as shown in Table 1 [27].

**Table 4:** Evaluation of in situ hybridisation (ISH) of PaBV-4 infected organs of four adult and two juvenile cockatiels.

Tissue							In situ Hybridisation
	B 0,1	C 0,1	C 1,0	D 1,0	J 8	J 10	Labelled Cells
**Nervous System**							
**Brain**	n/a	+(+)	+++	+(+)	+++	n/a	Neurons (incl. spinal ganglia), neuronal processes, glial cells, ependymal cells, mononuclear cells, vessel walls, meninges
**Spinal cord**	n/a	-	++	+	+(+)	n/a	
**N. ischiadicus**	n/a	-	-	n/a	n/a	n/a	-
**Eye**	n/a	++	+	(+)	++	n/a	Retinal cells, conjunctival epithelium, corneal epithelium, connective tissue, iris
**Digestive System**							
**Crop**	+	++	n/a	n/a	++	n/a	Ganglion cells, nerve fibres, fibrocytes, smooth muscle cells of all GIT layers, epithelium, mononuclear cells, vessel walls, adjacent fatty tissue
**Proventriculus**	+	++++	n/a	n/a	+++	n/a	
**Gizzard**	+	+++	n/a	n/a	+++	n/a	
**Intestine**	+	+++	n/a	n/a	++	n/a	
**Liver**	n/a	(+)	n/a	n/a	(+)	(+)	Vessel walls, fibrocytes
**Pancreas**	n/a	++	n/a	n/a	+	++	Exocrine cells, fibrocytes, vessel walls, mononuclear cells
**Respiratory System**							
**Trachea**	n/a	n/a	n/a	n/a	n/a	+(+)	Epithelium
**Lung**	n/a	+	n/a	n/a	+	+	Bronchial epithelium, smooth muscle cells, mononuclear cells, fibrocytes, vessel walls, pneumocytes, respiratory macrophages
**Urogenital System**							
**Kidney**	n/a	++	n/a	n/a	+(+)	++++	Tubular cells, Bowman’s capsule and mesangium of glomeruli, mononuclear cells, fibrocytes, vessel walls
**Ovary**	n/a	+++	n/a	n/a	n/a	n/a	Follicle epithelium, fibrocytes, smooth muscle cells
**Oviduct**	n/a	+++	n/a	n/a	n/a	n/a	Oviduct epithelium, fibrocytes, smooth muscle cells, mononuclear cells
**Testis**	n/a	n/a	n/a	n/a	+	+	Fibrocytes, smooth muscle cells, Sertoli cells, spermatogonia
**Other organs**							
**Adrenal gland**	n/a	n/a	n/a	+	n/a	n/a	Ganglion cells, mononuclear cells
**Heart**	n/a	+	n/a	n/a	+	++	Ganglion cells, nerve fibres, endocardium, smoot muscle cells of vessel walls, connective tissue, cardiomyocytes
**Spleen**	n/a	+	n/a	n/a	+	++	Mononuclear cells (lymphocytes, monocytes, reticular cells), fibrocytes, vessel walls
**Skeletal Muscle**	n/a	(+)	n/a	n/a	(+)	n/a	Vessel walls, nerve fibres
**Skin**	n/a	++	n/a	n/a	+(+)	n/a	Follicular epithelium, nerve fibres fibrocytes, smooth muscle cells, vessel walls, mononuclear cells, epidermal epithelium

n/a = not available or evaluable; Scoring system applicated as shown in Table 1 [27].

## Data Availability

Supplemental data presented in this study are available in the Supplemental Material Tables S1 and S2. Additionally, part of the data presented in this study are available in DOI: 10.1080/03079457.2020.1852177.

## Supplementary Materials

The following supporting information can be downloaded at: https://www.mdpi.com/article/10.3390/v14102181/s1, Table S1: Comparison of immunohistochemical and RT-qPCR evaluation of organs of 11 cockatiels infected with PaBV-4 as adults; Table S2: Comparison of immunohistochemical and RT-qPCR evaluation of organs of 11 cockatiels infected with PaBV-4 as juveniles.
