# Supplementary material for: Tissue Distribution of Parrot Bornavirus 4 (PaBV-4) in Experimentally Infected Young and Adult Cockatiels (Nymphicus hollandicus)
Source: Viruses. 2022 Oct 1;14(10):2181. doi: 10.3390/v14102181 (PMC9611548; doi:10.3390/v14102181)
Supplement: Supplementary file 1 [file viruses-14-02181-s001.zip › Table S1. Comparison of immunohistochemical and RT-qPCR evaluation of organs of 11 cockatiels infected with PaBV-4 as adults.pdf]

Table S1. Detailed immunohistochemical evaluation of organs of 11 cockatiels infected with PaBV-4 as adults (data generated as part of the here presented study) in comparison with RT-qPCR results (published in Gartner et. al, 2021)

	A 0,1		A 1,0		B 0,1		B 1,0		C 0,1		C 1,0		D 0,1		D 1,0		E 0,1		E 1,0		F 0,1	
	[a]IHC	[b]PCR	IHC	PCR	IHC	PCR	IHC	PCR	IHC	PCR	IHC	PCR	IHC	PCR	IHC	PCR	IHC	PCR	IHC	PCR	IHC	PCR
**Brain**	+	17,3*	++	13,01*	++	13,43*	++	12,69*	+++	13,21*	++	14,92*	++	16,21*	+	20,66*	+	13,45*	+++	12,69*	+++	15,54*
**Spinal cord**	++	14,29	++	15,52	+(+)	14,47	++	14,07	++	13,84	+	14,74	++	13,63	+	21,93	+	14,95	++	14,10	+++	13,80
**N. ischiadicus**	+	19,03	+	18,76	-	18,63	n/a	17,81	n/a	14,95	+	19,89	n/a	18,04	n/a	24,44	+	17,71	n/a	16,69	n/a	16,22
**Trachea**	-	n/a	-	n/a	-	n/a	n/a	n/a	-	n/a	-	n/a	-	n/a	-	n/a	-	n/a	-	n/a	-	n/a
**Lung**	-	29,68	-	16,25	+	25,21	(+)	19,94	(+)	15,45	-	26,47	+	17,62	-	31,97	+(+)	17,84	+	15,88	+	15,58
**Crop**	++	22,51	++	21,46	(+)	22,39	+	36,5	+	14,92	-	neg	++	22,17	+	23,08	++	17,2	+	16,59	+(+)	16,07
**Proventriculus**	+	23,01	(+)	17,57	+(+)	18,05	++	18,26	(+)	15,71	-	25,05	++	34,71	-	28,75	++	19,02	-	20,50	n/a	16,50
**Gizzard**	++	22,58	n/a	20,64	+(+)	21,36	++	36,83	n/a	16,85	-	27,14	-	18,02	+	24,15	+	20,53	-	18,53	n/a	14,93
**Intestine**	+	27,47*	+	19*	+(+)	20,56*	++	24,89*	+	14,11*	-	30,36*	+(+)	14,29*	+	26,27*	++	18,82*	+	16,65*	+(+)	13,42*
**Liver**	-	30,10	-	32,96	-	25,40	-	28,16	-	23,56	-	neg	-	25,98	-	neg	-	25,30	-	31,38	-	26,80
**Pancreas**	-	24,53	-	21,80	-	27,67	-	28,01	-	13,85	-	28,94	(+)	16,20	-	32,47	-	19,71	-	17,85	-	17,12
**Spleen**	-	25,17	+	20,39	-	24,87	-	28,36	+	19,07	-	26,28	+	15,71	-	33,07	+(+)	20,04	-	18,13	(+)	14,48
**Kidney**	-	28,05	-	21,94	-	25,98	-	23,13	++	15,52	-	29,15	++	15,36	-	31,65	+(+)	16,32	(+)	19,03	++	18,52
**Ovary**	-	18,15	n/a	n/a	-	21,57	n/a	n/a	+	18,41	n/a	n/a	+	13,39	n/a	n/a	+(+)	12,47	n/a	n/a	+	11,86
**Oviduct**	-	29,40	n/a	n/a	-	28,95	n/a	n/a	(+)	15,60	n/a	n/a	(+)	12,50	n/a	n/a	+(+)	15,38	n/a	n/a	(+)	11,59
**Testis**	n/a	n/a	-	26,09	n/a	n/a	-	19,34	n/a	n/a	-	22,80	n/a	n/a	-	28,34	n/a	n/a	n/a	14,16	n/a	n/a
**Adrenal gland**	++	15,07	+	15,44	+	16,46	n/a	13,88	n/a	12,49	+	14,93	-	12,42	+(+)	17,24	+	13,76	n/a	14,31	+++	12,13
**Eye**	-	19,77	+	13,60	+	14,11	+	14,05	++	16,49	-	21,34	-	14,18	-	26,53	+	14,24	+	14,71	+++	11,78
**Skin (Neck)**	-	26,23	+	21,40	+	21,04	-	18,91	n/a	13,01	-	23,44	-	14,99	-	34,96	+(+)	14,28	-	18,54	+	13,07
**Skin (Abdomen)**	-	26,19	+	21,81	+	36,02	-	20,74	+	12,60	-	21,27	-	16,01	-	28,15	+	13,28	-	16,89	+	13,69
**Heart**	-	30,52	(+)	23,79	(+)	23,00	(+)	21,04	+	21,60	(+)	33,65	+	19,17	(+)	32,62	+	21,07	(+)	26,41	+	34,22
**Skeletal Muscle**	-	34,82	-	25,93	(+)	26,69	-	35,18	-	41,06	-	neg	-	33,98	-	neg	+	39,45	-	35,82	(+)	31,51

[a] IHC: Immunohistochemistry for the detection of viral antigen in tissue applying an antibody directed against the bornaviral phosphoprotein (P); a scoring system applicated as shown in table 1: +++ = high no. of positive cells; ++ = moderate no. of positive cells; + = low no. of positive cells; (+) = questionable; - = no positive cells; n/a = not available (Petzold et al., 2019)

[b] PCR: real-time RT-PCR for the detection of viral RNA content in organ samples (Ct-values); n/a = not available; neg = negative (Gartner et al., 2021)

* Regarding brain and intestinal tissue, for a better comparability of results, the average Ct-value from cerebrum and cerebellum and large and small intestine, respectively is presented (Gartner et al., 2021)
