# Supplementary material for: Tissue Distribution of Parrot Bornavirus 4 (PaBV-4) in Experimentally Infected Young and Adult Cockatiels (Nymphicus hollandicus)
Source: Viruses. 2022 Oct 1;14(10):2181. doi: 10.3390/v14102181 (PMC9611548; doi:10.3390/v14102181)
Supplement: Supplementary file 1 [file viruses-14-02181-s001.zip › Table S2. Comparison of immunohistochemical and RT-qPCR evaluation of organs of 11 cockatiels infected with PaBV-4 as juveniles.pdf]

Table S2. Detailed immunohistochemical evaluation of organs of 11 cockatiels infected with PaBV-4 as juveniles (data generated as part of the here presented study) in comparison with RT-qPCR results (published in Gartner et. al, 2021)

	J 1		J 2		J 3		J 4		J 5		J 6		J 7		J 8		J 9		J 10		J 11	
	[a]IHC	[b]PCR	IHC	PCR	IHC	PCR	IHC	PCR	IHC	PCR	IHC	PCR	IHC	PCR	IHC	PCR	IHC	PCR	IHC	PCR	IHC	PCR
**Brain**	++	14,1*	++	11,47*	+	12,51*	+	13,59*	+	14,79*	+	11,47*	++	14,97*	+++	14,51*	+	13,25*	+++	14,36*	++	15,29*
**Spinal cord**	++	14,17	++	12,89	+	14,39	(+)	12,67	+	15,19	+	13,16	++	13,98	++	13,17	+	14,20	++(+)	16,33	++	14,09
**N. ischiadicus**	n/a	16,74	n/a	15,33	n/a	20,34	-	15,58	n/a	18,07	n/a	16,08	++	16,54	++	16,55	+	17,83	+	15,41	**n/a**	15,45
**Trachea**	-	n/a	-	n/a	-	n/a	-	n/a	-	n/a	-	n/a	-	n/a	+	n/a	n/a	n/a	+	n/a	+(+)	n/a
**Lung**	(+)	18,83	+	14,46	-	14,66	+	15,28	-	15,77	-	14,82	++	13,90	++	16,16	+	20,77	+(+)	16,36	+(+)	16,41
**Crop**	+	18,79	+	21,54	+	17,16	-	15,09	+	14,90	+(+)	20,96	+(+)	17,57	++	18,03	+	28,85	+(+)	16,07	+(+)	15,09
**Proventriculus**	+	16,99	+	12,72	+	14,37	(+)	16,15	+	14,25	++	30,98	+	15,33	++	18,82	+(+)	14,25	++	13,60	++	17,62
**Gizzard**	+	15,77	+	16,14	+	17,20	-	14,18	+	17,08	+(+)	20,17	+	15,83	++	18,01	+	16,22	+(+)	15,34	++	18,17
**Intestine**	+	15,67*	++	13,1*	+	14,41*	+	15,24*	+	15,62*	++	17,59*	+	17,1*	++	19,15*	+	16,01*	+(+)	13,92*	++	29,03*
**Liver**	-	27,05	-	31,44	-	25,31	-	25,97	-	27,58	-	24,51	-	30,72	-	22,48	-	25,12	-	25,70	-	25,13
**Pancreas**	(+)	16,62	(+)	15,96	-	15,65	+	14,73	-	15,49	(+)	16,21	(+)	17,55	+	17,00	-	16,73	-	14,69	-	18,10
**Spleen**	+	15,40	-	18,68	+	14,66	+	14,14	-	15,46	-	22,22	+	13,71	++	18,42	+	19,90	+(+)	13,66	+(+)	14,38
**Kidney**	+	15,81	+(+)	16,22	+	15,29	+	13,93	+	12,88	-	16,39	+	13,70	++	16,17	n/a	16,03	++	14,84	+(+)	14,72
**Ovary**	n/a	n/a	+(+)	11,05	+	11,31	+	10,42	n/a	n/a	-	13,19	+	12,60	n/a	n/a	n/a	14,74	n/a	n/a	+(+)	18,46
**Oviduct**	n/a	n/a	-	14,18	+	16,23	+	12,94	n/a	n/a	-	18,76	(+)	11,79	n/a	n/a	n/a	15,25	n/a	n/a	-	24,45
**Testis**	-	11,77	n/a	n/a	n/a	n/a	n/a	n/a	-	14,69	n/a	n/a	n/a	n/a	+	13,62	n/a	n/a	+(+)	12,59	n/a	n/a
**Adrenal gland**	n/a	13,84	+	11,99	+	13,30	+	11,22	-	14,24	n/a	14,28	++	13,45	+	12,71	+	13,76	+++	13,25	++(+)	22,87
**Eye**	n/a	16,26	+	12,86	+	12,80	-	13,02	-	15,26	(+)	12,08	+	17,20	+	15,63	-	13,60	+(+)	10,96	++(+)	22,81
**Skin (Neck)**	+	14,48	(+)	13,55	-	13,29	-	11,64	+	12,99	+	16,90	+	14,23	+	13,65	(+)	16,90	+(+)	11,50	+(+)	12,10
**Skin (Abdomen)**	+	14,40	-	14,39	-	13,97	-	10,88	+	14,09	+	20,00	+	14,26	+	14,87	+	16,01	+(+)	12,78	+(+)	12,74
**Heart**	+(+)	22,69	+	20,21	+	19,45	+	24,90	+	20,99	+	22,81	+	30,56	+	16,73	(+)	31,67	+(+)	20,29	+(+)	25,73
**Skeletal Muscle**	-	neg	-	neg	-	13,93	-	neg	(+)	36,65	+	29,23	-	neg	+	38,84	(+)	30,10	+	23,96	+	33,88

[a] IHC: Immunohistochemistry for the detection of viral antigen in tissue applying an antibody directed against the bornaviral phosphoprotein (P); a scoring system applicated as shown in table 1: +++ = high no. of positive cells; ++ = moderate no. of positive cells; + = low no. of positive cells; (+) = questionable; - = no positive cells; n/a = not available (Petzold et al., 2019)

[b] PCR: real-time RT-PCR for the detection of viral RNA content in organ samples (Ct-values); n/a = not available; neg = negative (Gartner et al., 2021)

* Regarding brain and intestinal tissue, for a better comparability of results, the average Ct-value from cerebrum and cerebellum and large and small intestine, respectively is presented (Gartner et al., 2021)
